# Microvesicles released from pneumolysin-stimulated lung epithelial cells carry mitochondrial cargo and suppress neutrophil oxidative burst

**DOI:** 10.1038/s41598-021-88897-y

**Published:** 2021-05-05

**Authors:** E. Letsiou, L. G. Teixeira Alves, D. Fatykhova, M. Felten, T. J. Mitchell, H.C. Müller-Redetzky, A. C. Hocke, M. Witzenrath

**Affiliations:** 1 Division of Pulmonary Inflammation, and Department of Infectious Diseases and Respiratory Medicine, Charité – Universitätsmedizin Berlin, corporate member of Freie Universität Berlin and Humboldt-Universität zu Berlin, 10117 Berlin, Germany; 2 Division of Pulmonary, Critical Care, Sleep and Allergy, Department of Medicine, University of Illinois at Chicago, Chicago, IL 60612 USA; 3 Institute of Microbiology and Infection, College of Medical and Dental Sciences, University of Birmingham, Birmingham, B15 2TT UK; 4 German Center for Lung Research, (DZL), Berlin, Germany

**Keywords:** Mechanisms of disease, Infection, Cell biology

## Abstract

Microvesicles (MVs) are cell-derived extracellular vesicles that have emerged as markers and mediators of acute lung injury (ALI). One of the most common pathogens in pneumonia-induced ALI is *Streptococcus pneumoniae* (Spn), but the role of MVs during Spn lung infection is largely unknown. In the first line of defense against Spn and its major virulence factor, pneumolysin (PLY), are the alveolar epithelial cells (AEC). In this study, we aim to characterize MVs shed from PLY-stimulated AEC and explore their contribution in mediating crosstalk with neutrophils. Using in vitro cell and ex vivo (human lung tissue) models, we demonstrated that Spn in a PLY-dependent manner stimulates AEC to release increased numbers of MVs. Spn infected mice also had higher levels of epithelial-derived MVs in their alveolar compartment compared to control. Furthermore, MVs released from PLY-stimulated AEC contain mitochondrial content and can be taken up by neutrophils. These MVs then suppress the ability of neutrophils to produce reactive oxygen species, a critical host-defense mechanism. Taken together, our results demonstrate that AEC in response to pneumococcal PLY release MVs that carry mitochondrial cargo and suggest that these MVs regulate innate immune responses during lung injury.

## Introduction

Community-acquired pneumonia (CAP) causes considerable morbidity and mortality despite existing antibiotic therapies and preventive vaccination strategies^1^. The most prevalent bacterial pathogen causing CAP is the Gram-positive bacterium, *Streptococcus pneumoniae* (Spn). Patients with pneumococcal pneumonia are at high risk for progression to life-threatening conditions such as sepsis and acute respiratory distress syndrome (ARDS) despite antibiotic therapy^2^. Notwithstanding decades of investigations on host–pathogen interactions, further research is needed in order to identify new approaches against the development of ARDS in pneumonia.

Numerous studies have demonstrated that Spn mediates its damaging effects by releasing the pore-forming toxin, pneumolysin (PLY), during bacterial autolysis^3^. PLY monomers bind to cholesterol on cellular membranes and subsequently oligomerize to form large pores. PLY-induced pore formation results in intracellular calcium increase and induction of multiple cellular responses, including cell lysis and death at high doses^4^. PLY has a direct role in pneumonia pathogenesis and CAP complications, such as acute lung injury (ALI)/ARDS^3,5^. It causes lung endothelial and epithelial barrier disruption, immune system dysregulation, and facilitation of Spn colonization^6–8^, but the cellular mechanisms underlying these PLY-induced events are incompletely understood.

Upon release into the airspace, PLY targets alveolar cells including the epithelium. Previous studies from our group and others have demonstrated that PLY initiates numerous inflammatory responses in alveolar epithelial cells (AEC) leading to cellular dysfunction, such as mitochondrial injury, necroptosis and DNA damage^9–11^, inflammatory cytokine release^12^, and dysfunction of the epithelial sodium channels^8^. Recently it was shown that PLY could also serve as a strong stimulant for extracellular vesicle (EV) production^13–15^.

EVs are small membrane-derived vesicles released from cells under normal conditions, or upon activation and cell death (e.g. apoptosis, necroptosis, pyroptosis)^16,17^. Depending on their physical characteristics, EVs are classified into different categories including microvesicles (MVs), which are medium/large vesicles (size range of 0.1–1 μm), and exosomes, which are small vesicles (~ 30–150 nm). Recent studies by our group and others have identified MVs as important mediators of ALI^18–23^. MVs consist of lipids, proteins, and nucleic acids, and their content depends on the cellular origin as well as the conditions that stimulated their biogenesis and release^17^. They play a major role in mediating cellular cross-talk, which is primarily attributed to their capacity to transfer their specific molecular cargo to recipient cells. Despite the increasing number of studies exploring MVs in the field of lung diseases^24^, the role of MVs of alveolar epithelial origin in the context of pneumococcal pneumonia-induced ALI is poorly understood. Interestingly, our previous studies showed that AEC exposed to PLY release mitochondrial DNA (mtDNA) extracellularly, within the MV fraction^9^. Based on these previous observations and existing literature, we hypothesized that lung alveolar epithelial cells in response to PLY release increased amounts of MVs carrying mitochondrial cargo that can be transferred to recipient neutrophils to regulate their immune functions. To test this hypothesis, we performed studies to (a) determine the effects of PLY on MV release from lung epithelial cells, (b) explore the mitochondrial content of PLY-induced MVs, and (c) examine the functional role of PLY-induced MVs on neutrophils, which are relevant immune cells that transmigrate to the alveolar space rapidly upon infection.

## Results

### Characterization of microvesicles released by PLY-treated alveolar epithelial cells

**Figure 1:**
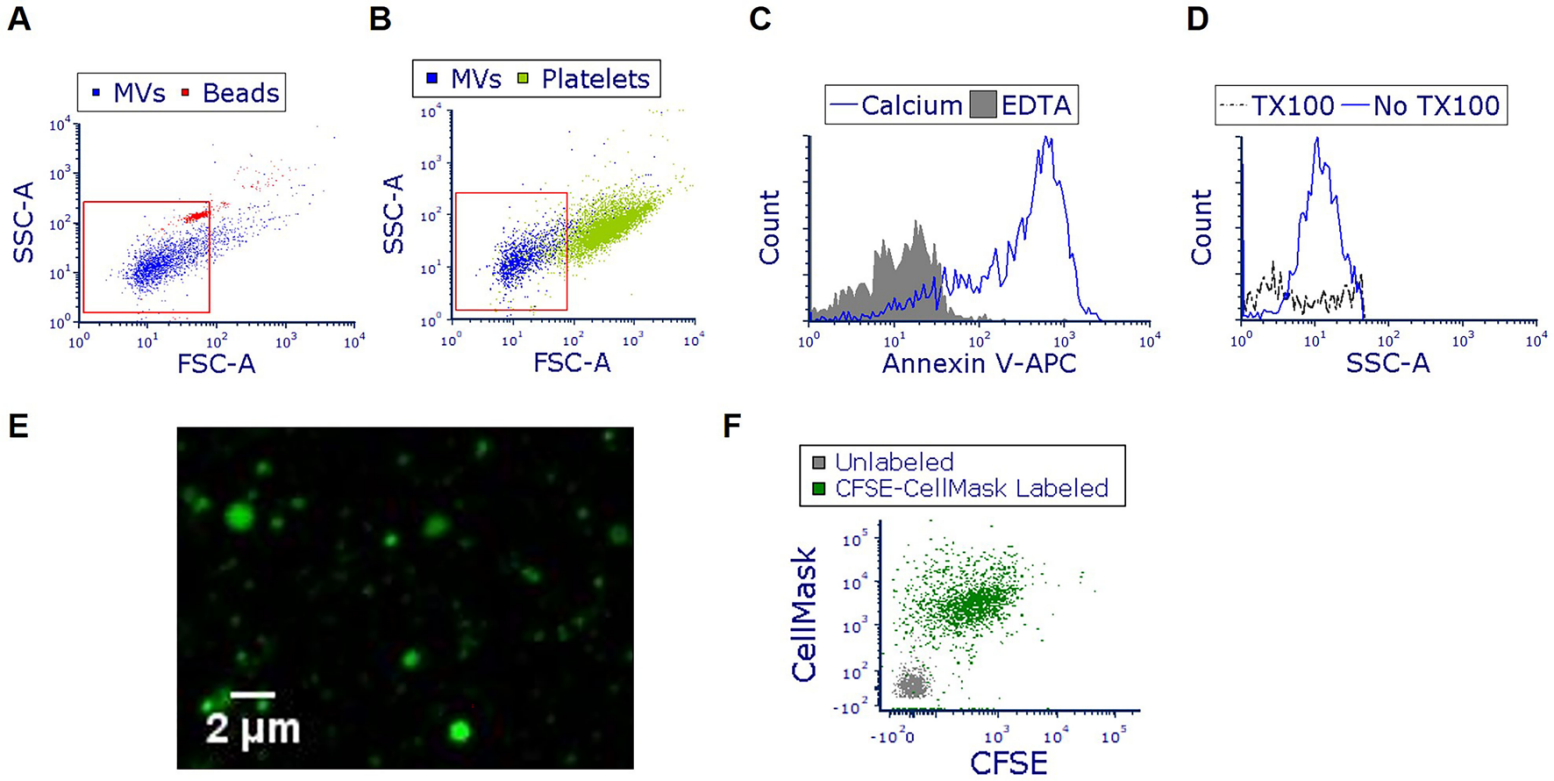
Characterization of microvesicles (MVs) produced from pneumolysin (PLY)-treated alveolar epithelial cells. A549 cells were treated with PLY (100 ng/ml). After 4 h, conditioned media was collected and MVs were isolated and characterized. (**A,B**) Representative FACS analysis (overlay dot blots) of isolated vesicles (blue dots), and of (**A**) 1 μm beads (red dots) or (**B**) human platelets (green dots). (**C**) MVs were stained with annexin V-APC in the presence or absence of calcium and analyzed by FACS. (**D**) MVs were treated with Triton-X 100 and analyzed by FACS. (**E**) CFSE-labeled MVs (green) were visualized using confocal microscopy. (**F**) MVs were labeled with CFSE (FITC) and CellMask (APC) and analyzed by FACS. In the representative dot blot, MVs labeled with CFSE and CellMask are shown in green. Grey dots represent unstained vesicles (background staining).

A549 were treated with PLY and MVs were isolated from the conditioned media after 4 h. Using FACS, we observed that the majority of isolated vesicles are < 1 μm (Fig. 1A,B), annexin V positive (annexin V +) (Fig. 1C), and sensitive to detergents (Fig. 1D). As seen in Fig. 1E (and Suppl. Fig. S1a), MVs can be visualized by confocal microscopy after labeling with cell permeable CFSE, demonstrating that they are heterogeneous in size. Finally, MVs are positive not only for CFSE, but also for CellMask, a plasma membrane dye (Fig. 1F). As expected, CFSE-labeled MVs are also annexin V positive (Suppl. Fig. S1b). These staining combinations provide further evidence for the vesicular nature of these isolated particles.

### Quantification of microvesicles released by PLY-treated alveolar epithelial cells

**Figure 2:**
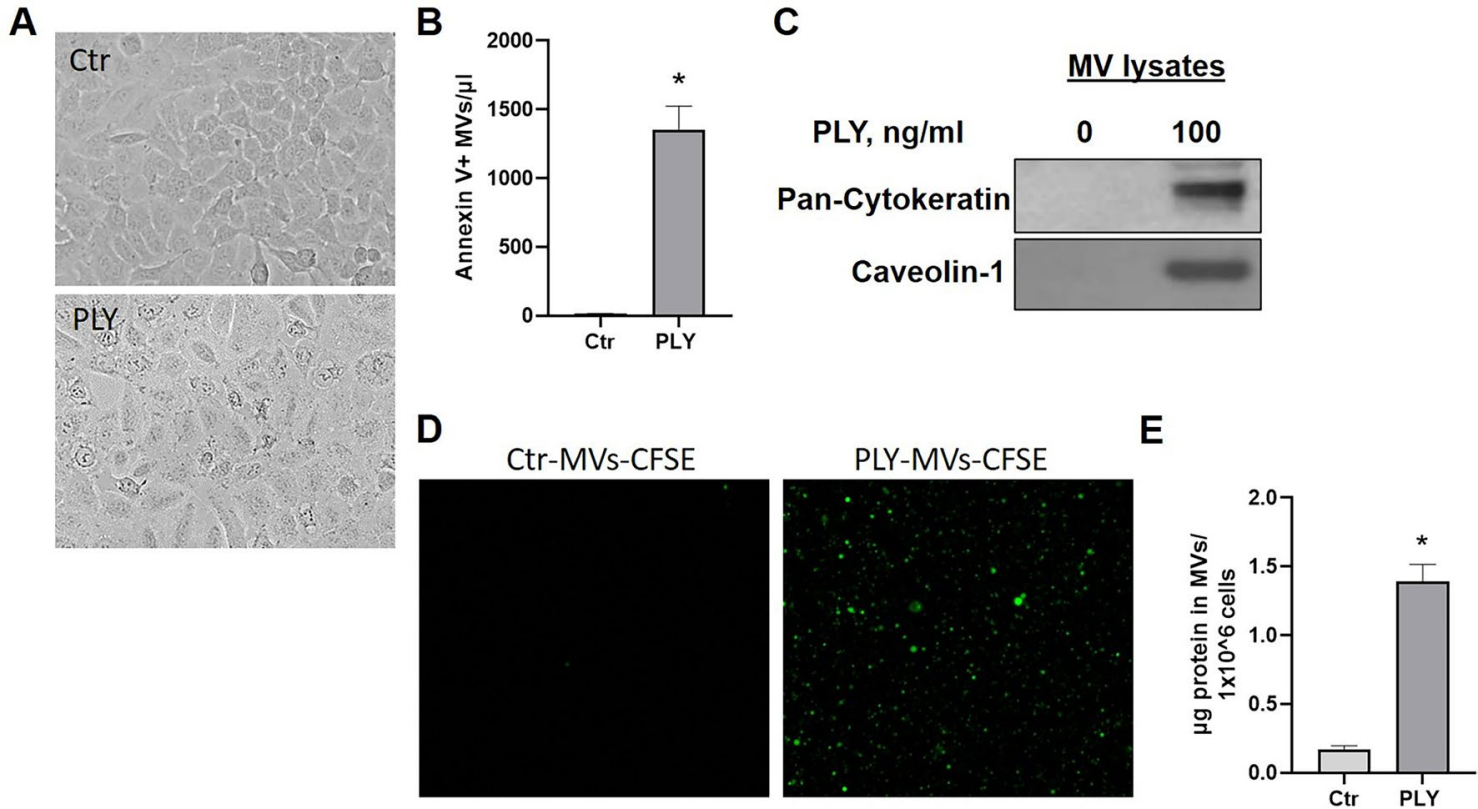
Pneumolysin (PLY) induces the release of alveolar epithelial microvesicles (MVs). A549 cells were treated with PLY (100 ng/ml) or PBS (Ctr) for 4 h. (**A**) Bright field images of cells 4 h after treatment. (**B**) Annexin V-positive ( +) MVs were quantified using FACS. N = 9, p < 0.0001 (upaired t-test). (**C**) MV lysates were probed with pan-cytokeratin and caveolin-1 antibodies. Depicted are representative blots (cropped images). (**D**) CFSE-MVs from Ctr or PLY-treated cells were visualized using confocal microscopy. (**E**) Protein amount in MVs derived from Ctr or PLY-treated cells. N = 9, *p < 0.0001 (unpaired t-test).

Next, we compared the production of MVs from control and PLY-treated cells. As depicted in Fig. 2A, at these experimental conditions (PLY 100 ng/ml 4 h), cells remain attached to the culture dish. Higher concentrations (> 200 ng/ml, 4 h) caused considerable cell detachment. For this reason, and consistent with concentrations used in other studies in the field^9,11^, we used 100 ng/ml of PLY to stimulate the cells. We then quantified the MV production by measuring the number of annexin V-positive MVs by FACS and observed a tremendous increase in MV release from PLY-treated cells compared to control (Fig. 2B). Consistent with this, high protein levels of pan-cytokeratin (epithelial marker) and Cav-1 (common MV protein) were detected by immunoblotting in MVs from PLY-treated cells (Fig. 2C). Similarly, a large amount of CFSE-MVs were detected in the media of PLY-treated cells compared to untreated cells, in which the CFSE signal was considerable lower (Fig. 2D). Isolated MVs from PLY-treated cells have much higher total protein amount than control, further confirming an increased production of MVs after PLY treatment (Fig. 2E).

### Epithelial MV production in a human lung tissue model

**Figure 3:**
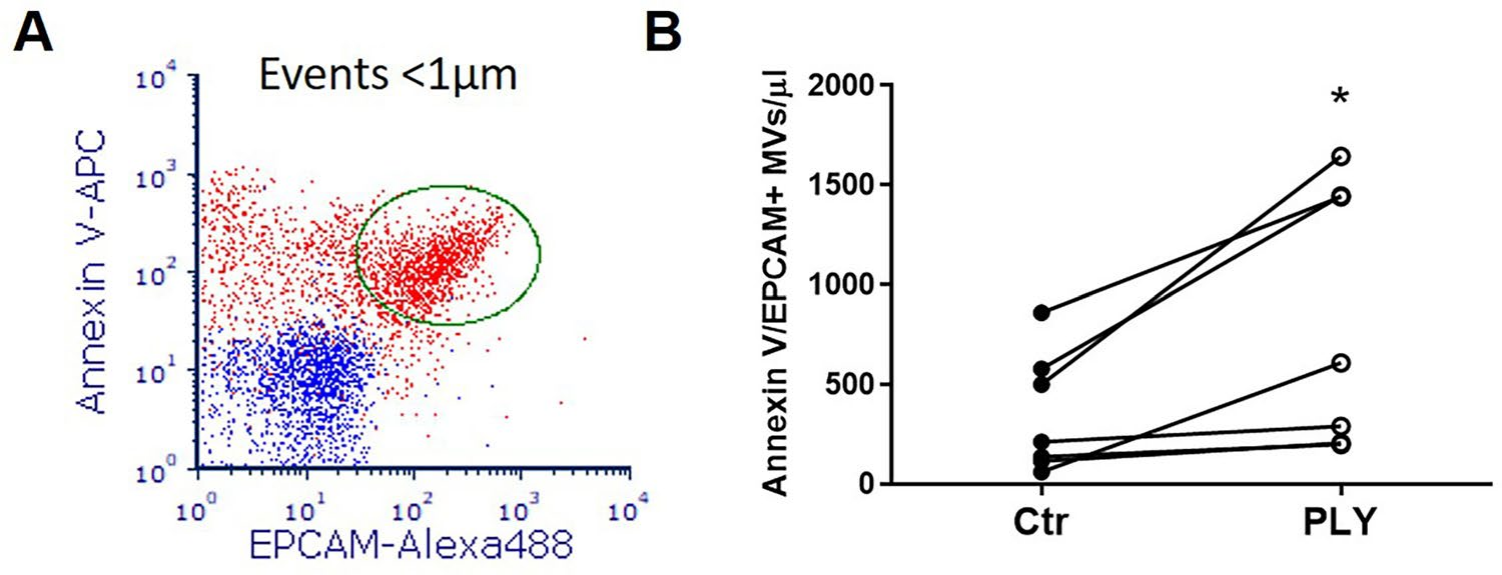
Ex vivo production of lung epithelial microvesicles (MVs). Human lung specimens (pieces from the same lung) were stimulated with PLY (5 μg/ml) or PBS (Ctr). MVs were isolated from the conditioned media 8 h later and analyzed by FACS. (**A**) Representative overlay dot blot of MVs from PLY-treated human lungs. Blue dots represent MVs double stained with isotype and annexin V in the presence of EDTA (negative staining). Red dots represent MVs double stained with annexin V-APC and EPCAM-Alexa488. Epithelial MVs (double positive events) are gated by the ellipse gate (green). (**B**) Graph depicts the quantification of annexin V/EPCAM double positive MVs. Each line represents data derived from different lung specimens (differentially treated) from the same patient. N = 7, *p < 0.05 (paired t-test)**.**

To examine whether lung epithelial cells can produce MVs when they are embedded in their original 3D tissue architecture, additional experiments were performed in human lung tissue explants^9,25^. Figure 3A depicts the gating strategy to identify epithelial MVs (annexin V/EPCAM double positive) in human lung tissue supernatant. Similar to the in vitro findings, we demonstrate a significant increase in ex vivo production of MVs of epithelial source after PLY challenge (p < 0.05) (Fig. 3B).

### Spn induces MV release via PLY

**Figure 4:**
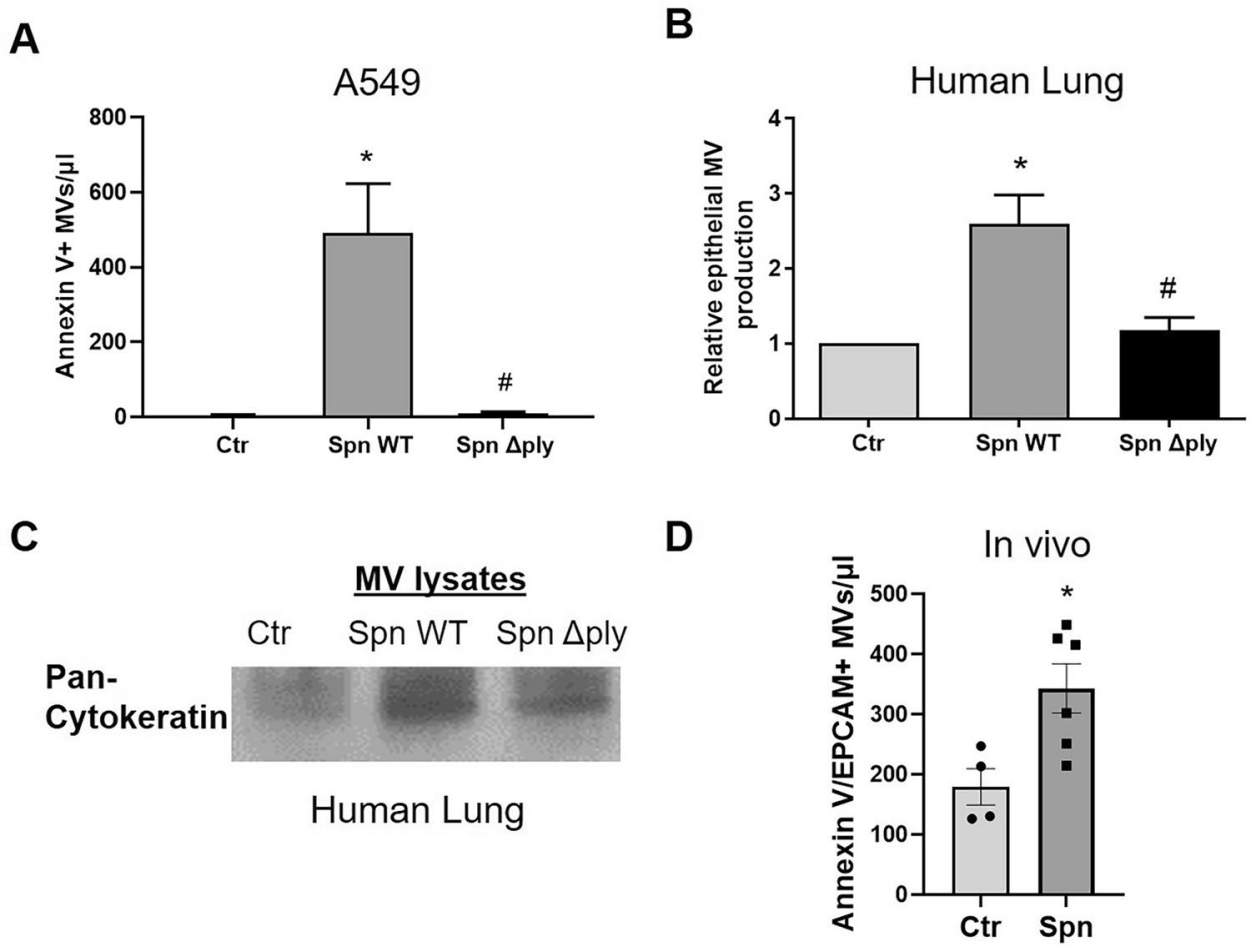
Streptococcus pneumoniae (Spn) induces epithelial microvesicle (MV) production via pneumolysin. (**A**) A549 cells were treated with Spn WT, Spn *Δply* (MOI 50), or PBS (Ctr) for 4 h and MVs were isolated from conditioned media. Annexin V + MVs were quantified by FACS. N = 4 *p < 0.01 vs Ctr, #p < 0.01 vs Spn Δ*ply* (One-way Anova). (**B,C**) Human lung specimens (pieces from the same lung) were treated with Spn WT, Spn *Δply *(1 × 10^6^ CFU/ml), or PBS. (**B**) Graph depicts the relative amount of annexin V/EPCAM double positive MVs (normalized to Ctr). N = 6, *p < 0.05 vs Ctr, #p < 0.05 vs Spn ΔPly (One-way Anova). (**C**) MV lysates from human lung tissue supernatants probed for pan-cytokeratin (epithelial marker). Depicted is a representative blot (cropped image). (**D**) MVs of epithelial origin (annexin V/EPCAM double positive) were quantified in BAL of mice treated intranasally with Spn or PBS (Ctr). Each mouse is represented by a dot. N = 4–6, *p < 0.05 (unpaired t-test).

To explore the potential contribution of other pneumococcal virulence factors on MV release, A549 were challenged with live bacteria (Spn WT vs Δ*ply*). Spn WT caused a significant release of annexin V-positive MVs (p < 0.01), however, the production of MVs after treatment with the PLY-deficient strain (Δ*ply*) was minimal (Fig. 4A). Human lung tissue in response to Spn WT produced an increased amount of epithelial MVs (double positive annexin V/EPCAM MVs) compared to untreated lung (Fig. 4B). However, MV production was significantly decreased in lung tissues stimulated with the PLY-deficient Spn strain (Fig. 4B). Consistent with this, MVs derived from Spn WT-stimulated human lung had increased levels of pan-cytokeratin compared to untreated or Spn Δ*ply*-treated (Fig. 4C). Taken together, these data indicate that pneumococcus stimulates the host to produce MVs via PLY-dependent mechanisms.

### Spn induces epithelial MV release into the alveolar space

48 h post Spn infection in vivo, a time point at which there is severe lung injury^26,27^, we observed a significant increase of annexin V/EPCAM + MVs by almost two-fold in the BAL of infected mice compared to controls (p < 0.05) (Fig. 4D).

### MV release is mediated by pore formation and calcium influx induced by PLY

**Figure 5:**
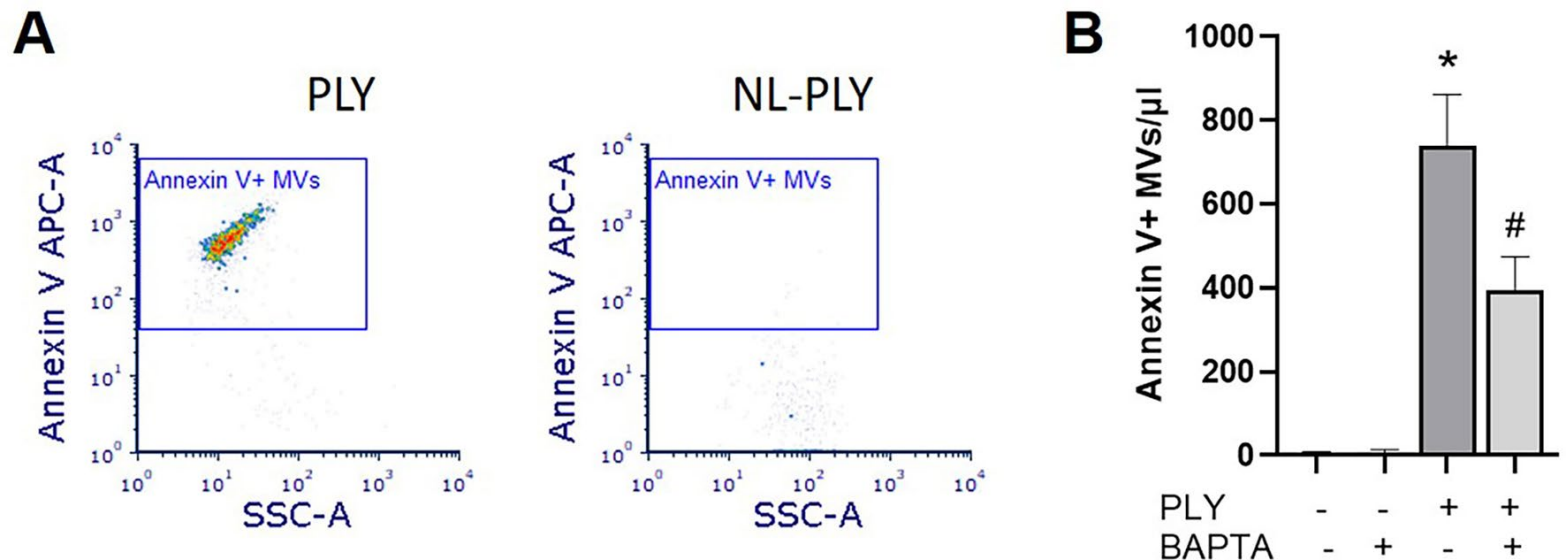
Pneumolysin (PLY) induces alveolar epithelial microvesicle (MV) release through pore formation and calcium influx. (**A**) A549 cells were treated with PLY or non-lytic (NL)-PLY (100 ng/ml) for 4 h and isolated MVs were analyzed by FACS. Depicted are representative dot blots of MVs after staining with annexin V. Positive events are indicated in the blue border gate. (**B**) A549 cells were pre-treated with BAPTA-AM (30 μΜ) for 1 h and then treated with PLY (100 ng/ml, 1 h). MVs were quantified by FACS after staining with annexin V. N = 3, *p < 0.001 vs Ctr, #p < 0.05 vs PLY (One-way Anova).

To explore the mechanisms by which PLY induces MV release, we employed a non-lytic (NL) PLY mutant that binds to cells but does not cause pores^28^. Our data demonstrate that annexin V + MV production is diminished in cells treated with NL-PLY compared to PLY (Fig. 5A). Next, we determined the role of calcium in MV production. Intracellular calcium release is a major stimulant for MV release. As expected, reducing the intracellular calcium concentration with a well-known calcium chelator, BAPTA-AM, caused a significant decrease by 47% in MV production after PLY treatment (p < 0.05) (Fig. 5B).

### PLY-stimulated alveolar epithelial cells release MVs enriched in mitochondrial cargo

**Figure 6:**
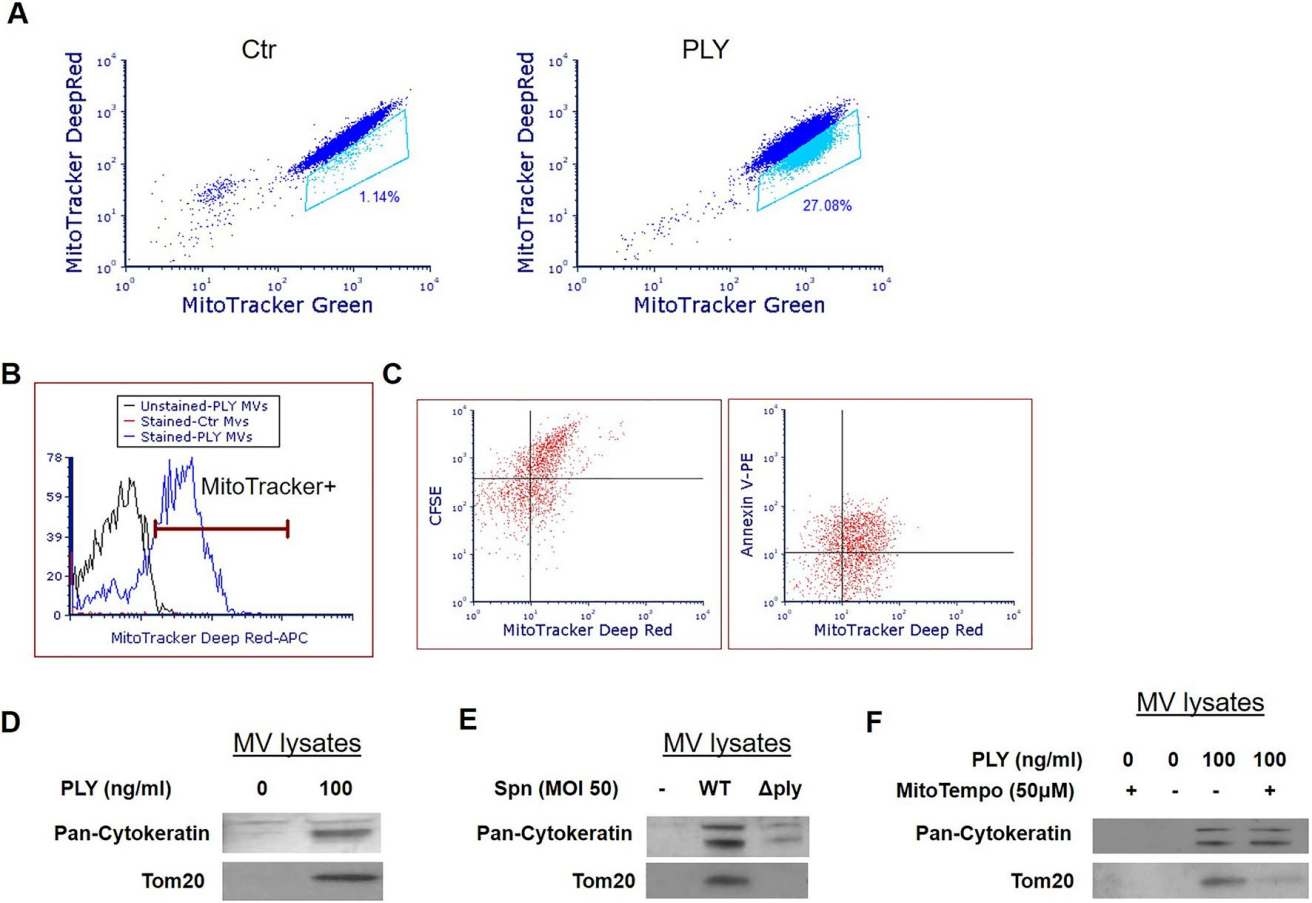
Pneumolysin (PLY) triggers the release of microvesicles (MVs) carrying mitochondrial cargo. (**A**) MitoTracker Deep Red and Green staining of A549 cells after treatment with PLY (100 ng/ml, 4 h) or PBS (Ctr). Gated cells stain less brightly with Mitotracker Deep Red after PLY indicative of loss of membrane potential. (**B,C**) MitoTracker Deep Red-labeled A549 were challenged with PLY (100 ng/ml, 4 h) or PBS. MVs were analyzed by FACS (**B**) or after staining with (**C**) CFSE or annexin V-PE. (**D–F**) A549 were challenged with (**D**) PLY (100 ng/ml, 4 h), (**E**) Spn WT vs Δ*ply* (MOI 50, 4 h), or (**F**) pre-treated with MitoTempo (50 μM) or DMSO prior to PLY (100 ng/ml, 4 h). MVs were isolated from the conditioned media and analyzed by immunoblotting. Depicted are representative blots of Tom20 (mitochondrial protein) and pan-cytokeratin (cropped images). Experiments were performed 3 or more independent times.

We have recently shown that Spn and PLY induce mitochondrial dysfunction in AEC, as evidenced by the increased mitochondrial calcium influx and loss of mitochondrial membrane potential, mitochondrial fragmentation, reduction of cytosolic and mitochondrial ATP levels, and mtDNA release within MVs^9^. In addition, recent studies suggest that EVs can be carriers of mitochondria^29–31^. Based on these observations, we next examined whether PLY-induced MVs carry mitochondria. Initially, we aimed to confirm that PLY induces mitochondrial dysfunction in our experimental settings. A549 were co-stained with the mitochondrial dyes MitoTracker- Green (localizes to mitochondria independent of mitochondrial membrane potential) and -Deep Red (mitochondrial stain that is more sensitive to polarization) before PLY treatment, and mitochondrial membrane depolarization was assessed as described before^32^. As depicted in Fig. 6A, in response to PLY, a portion of cells (gated) depolarize as indicated by the reduction in the MitoTracker Deep Red signal (Fig. 6A).

To explore the mitochondrial cargo within the MVs, MVs from MitoTracker Deep Red-labeled A549 (PLY treated) were isolated and analyzed (Fig. 6B) or co-stained with CFSE or annexin V-PE (Fig. 6C). As depicted in Fig. 6B, PLY-triggered AEC-MVs were enriched in mitochondrial dye. Importantly, MitoTracker Deep Red-positive MVs were also CFSE or annexin V positive, indicating the presence of mitochondria within the MVs (Fig. 6C). Note, that unstained and single stained populations were processed in parallel to establish the gating strategies (draw of quadrants), and the upper right gates indicate MVs double positive for MitoTracker Deep Red and CFSE or annexin V. Consistent with this, PLY-MVs were enriched in Tom20, a specific mitochondrial protein (Fig. 6D). Moreover, substantial higher levels of Tom20 were detected in MVs from Spn-treated cells compared to control or Spn Δ*ply*-treated (Fig. 6E). Interestingly, A549 cells (PLY stimulated) pretreated with MitoTempo, a mitochondrial-specific ROS scavenger that inhibits mitochondrial dysfunction^33,34^, produce MVs that express less Tom20 compared to MVs from vehicle-treated cells (Fig. 6F). MV pan-cytokeratin levels that reflect the total vesicle levels are unchanged after MitoTempo treatment of the parent cells. These data suggest that mitochondrial ROS scavenging in the parent cell results in the production of MVs with reduced mitochondrial content.

### PLY-induced alveolar epithelial MVs are taken up by human neutrophils and alter their function

**Figure 7:**
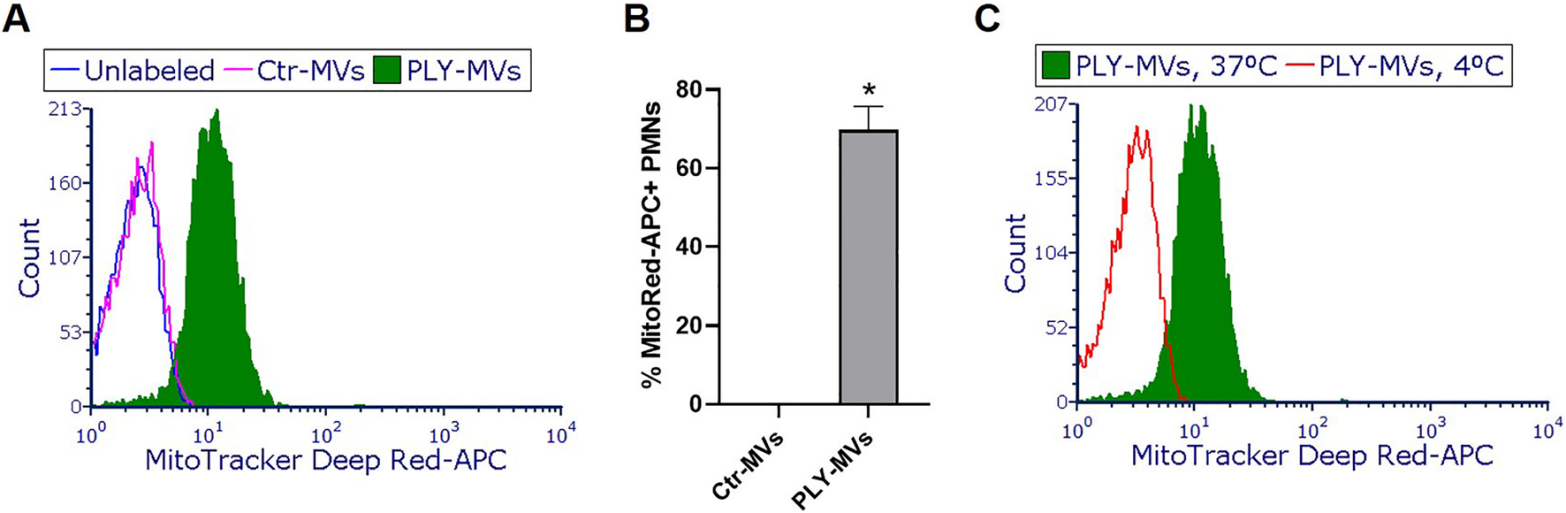
Microvesicles (MVs) released from pneumolysin (PLY)-treated lung alveolar epithelial cells are taken up by neutrophils. Human neutrophils (PMNs) were incubated with Ctr- or PLY-MitoTracker Deep Red- MVs [MVs were isolated from MitoTracker Deep Red-labeled A549 cells after treatment with PLY (100 ng/ml) or PBS- control (Ctr) at 37 °C or 4 °C for 1 h. PMNs were then analyzed by FACS for MitoTracker Deep Red signal. (**A**) Representative histogram of live PMNs (CD11b positive/7-AAD negative) treated with unlabeled MVs, Ctr- or PLY- MVs, at 37 °C. (**B**) Quantification of the percentage of MitoTracker Deep Red positive PMNs, n = 4, *p < 0.0001 (Mann–Whitney test, unpaired). (**C**) Representative histogram of live PMNs (CD11b positive/7-AAD negative) treated with PLY-MVs at 37 °C or 4 °C.

Having demonstrated that epithelial MVs released after PLY exposure carry mitochondrial cargo, we next assessed whether these MVs have a biological role. Towards this, we explored the interaction of alveolar epithelial MVs with neutrophils. During bacterial lung infection, neutrophils are recruited into the lungs to fight the invading pathogens and become the prominent cell type in the alveolar space. Therefore, neutrophils represent host target candidates for the MVs released from the alveolar epithelium. To investigate their interaction, MVs derived from MitoTracker Deep Red- labeled A549 untreated or PLY-treated were incubated with neutrophils, and their uptake was assessed by FACS. As seen in Fig. 7A,B, a significant portion of PMNs (CD11b positive/7-AAD negative gated) are positive for MitoTracker Deep Red after incubation with PLY-MVs but not Ctr or unlabeled MVs, suggesting that PMNs and MVs interact (p < 0.0001). We next assessed whether the MVs are taken up by PMNs or are surface-bound. For this, PMNs were incubated with PLY-MVs at 37 or 4 °C, as it has been previously shown that at 4 °C, MVs bind to cells but cannot be internalized^35^. We observed that MV uptake was temperature dependent, as PMNs were fluorescent only at 37 °C and not at 4 °C (Fig. 7C), suggesting that MVs-carrying mitochondrial cargo are internalized by PMNs.

**Figure 8:**
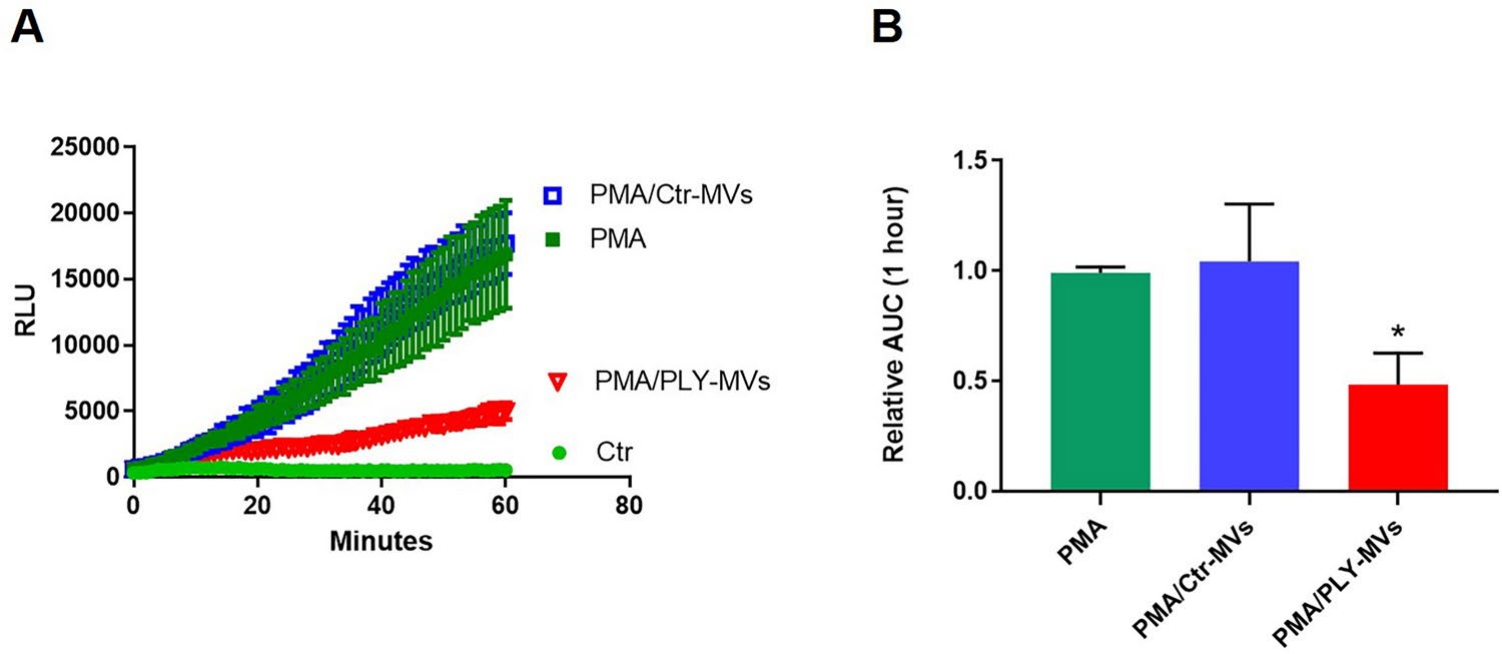
Effect of microvesicles (MVs) on reactive oxygen species (ROS) production from neutrophils. ROS production was measured in neutrophils (PMNs) primed with PMA (0.5 nM) and after stimulation with MVs isolated from control or PLY-treated A549 cells. ROS production was also measured in Ctr and PMA only treated PMNs (**A**) Representative kinetics of ROS production over a period of 1 h. (**B**) Quantification of ROS production. N = 7, *p < 0.001 vs PMA and PMA/Ctr-MVs (One-way Anova).

We then examined whether PLY-induced MVs can affect PMN functions. It is well known that neutrophils upon recruitment into the alveolar space produce ROS, as a defensive mechanism against pathogens. Therefore, we examined the effects of MVs on ROS production. Interestingly, we found that PMNs treated with MVs from PLY-derived lung epithelial cells had impaired ability to produce ROS after PMA challenge, while Ctr-MVs had no effect (Fig. 8A,B).

## Discussion

In this study, we demonstrate a new mechanism of host–pathogen interaction upon *Streptococcus pneumoniae* lung infection. We have found that Spn stimulates lung epithelial cells to release high amounts of MVs, a response dependent on the pneumococcal virulence factor PLY. Interestingly, MVs produced from PLY-stimulated alveolar epithelial cells contain mitochondrial cargo, which is taken up by neutrophils. This pathogen-stimulated interaction results in suppressed ROS production from activated neutrophils. Our study is novel in several important ways. First, this is the first study to perform a careful analysis of MV production from AEC in response to Spn and its major exotoxin PLY. Second, to our knowledge this is the only study showing that cells injured by bacteria and their products secrete MVs that carry mitochondrial cargo. Finally, this study demonstrates for the first time a PLY- stimulated cross-talk between AEC and neutrophils through MVs.

Emerging evidence suggests that extracellular vesicles are critical contributors to the pathogenesis of multiple disease processes, including pulmonary-related diseases^23,24^. Both the numbers and composition of EVs increase in the circulation and biological fluids during inflammatory states, reflecting specific pathophysiological mechanisms triggering their production and release. In the current study, we explored the production and role of lung epithelial MVs released upon Spn lung infection. Pulmonary pneumococcal infections are associated with severe complications such as the development of ALI/ARDS. PLY, an Spn exotoxin, is responsible for many of the deleterious effects of Spn^3,5^. We found that Spn in a PLY-dependent manner causes a significant production of MVs in alveolar epithelial cells, and we confirmed these data using a human lung tissue ex vivo model. Our data agree with recent reports demonstrating that PLY causes EV release. Wolfmeier et al. reported that HEK 293 cells exposed to PLY release vesicles of less than 1 μm^13^. Interestingly, the same study showed that these vesicles carry pneumolysin pores, and that this serves as a cell repair mechanism since it can eliminate PLY-induced pores from the affected cells. A more recent study demonstrated a PLY-dependent release of MVs from immune cells^14^. Similar to PLY, other bacterial toxins and bacteria have been found to trigger MV release in multiple cell types^36–38^.

Using a murine pneumococcal infection-induced ALI model, we demonstrated an increased epithelial MV release in the alveolar compartment. An elevation in the number of epithelial-derived MVs in BAL of ALI mice has been previously suggested by different groups. Lee et al. detected increased levels of MVs of epithelial source in BAL from mice challenged with hyperoxia and acid^20^. In contrast, exposure of mice to Pseudomonas did not result in epithelial MV production^20^. In mice treated with bacterial LPS, a common ALI model, data regarding epithelial MV production are contradictory^19,20^. Therefore, whether lung epithelial MV production is specific to Gram-positive or negative pulmonary infections warrants further investigation. Interestingly, increased alveolar epithelial EVs were observed in the edema fluid of ARDS patients^39^, although the cause of ARDS in these patients was not specified.

Regarding the mechanisms underlying MV production during Spn infection, our data demonstrate that PLY is the critical pneumococcal virulence factor that is responsible. WT Spn resulted in a significant release of MVs from AEC (Fig. 4); however, MV production was significantly diminished after exposure to an Spn mutant strain that does not express PLY. These data strongly suggest that PLY is the major Spn virulence factor involved in MV release. Furthermore, we observed that a PLY mutant protein that binds to cells but does not form pores failed to stimulate MV production from AEC (Fig. 5A). One explanation for this observation is that MV release is a calcium-dependent process^40,41^, consistent with the fact that PLY causes a significant calcium influx due to its pore-formation activity^9^. In the presence of a calcium chelator, PLY-induced MV production is significantly decreased in our model, although it was not completely inhibited (Fig. 5B), as other studies have shown^42^. Therefore, although these data suggest that PLY-induced MV release is at least partially dependent on calcium, calcium independent mechanisms cannot be excluded, as suggested elsewhere^43^.

In our previous study, we demonstrated that in response to PLY, AEC undergo increased mitochondrial fragmentation, reduction in ATP release, and increase of extracellular mtDNA, all signs of mitochondrial dysfunction^9^. Moreover, we found mtDNA within the MV fraction. Here, we expand these findings to demonstrate that MVs triggered by PLY are also carriers of mitochondria. Consistent with this, the presence of mitochondria in EVs generated under stressed conditions has been described by others. Platelets, astrocytes, and monocytes upon activation, macrophages that undergo necroptosis, airway exosomes, and apoptotic neuronal and glial cells, have all been reported to release EVs containing mitochondria^29–31,44–46^.

In agreement with our prior study showing mitochondrial dysfunction in PLY-treated cells^9^, other recent studies have demonstrated increased mitochondrial ROS (mtROS) production in PLY-treated lung endothelial cells and macrophages^47,48^. Increased mtROS levels can negatively affect mitochondrial function by opening of the mitochondrial permeability transition pores, which in turn induce mitochondrial depolarization and swelling and impaired mitochondrial activities^49^. An mtROS scavenger, MitoTempo, inhibits mitochondrial dysfunction and protects against injury after PLY and other stimuli in several cell types^33,34,48,50^. In our study, we demonstrated that mtROS inhibition in PLY-stimulated A549 cells results in the release of MVs that carry less mitochondrial content compared to MVs derived from control cells (Fig. 6F). These data suggest that scavenging mtROS interferes with the packaging of mitochondria within the MVs. This is consistent with a recent study showing reduced pro-inflammatory mitochondrial cargo in extracellular vesicles released from MitoTempo-treated monocytes^30^. One possible explanation is that mitochondrial dysfunction leads to accumulation of dysfunctional mitochondria, which in addition to internal elimination (e.g. through mitophagy), can also be eliminated by secretion within EVs. This hypothesis is supported by a recent study showing that mesenchymal cells undergoing oxidative stress and mitochondrial dysfunction secrete depolarized mitochondria within EVs and outsource mitophagy to macrophages^51^. In this process, mitochondrial protective agents (such as mtROS inhibitors) can reduce the accumulation of dysfunctional mitochondria, and therefore less mitochondrial cargo is present within the secreted vesicles.

Mitochondrial cargo not only is released extracellularly within EVs but can be transferred to target cells to alter their functions. For example, mitochondria from airway myeloid-derived regulatory cells are transferred via exosomes to T cells where they co-localize with the mitochondrial network of T cells and generate reactive oxygen species^29^. In another study, LPS-treated monocytes produced MVs rich in mitochondrial content that activated proinflammatory signaling in endothelial cells^30^. Consistent with these studies, we found that AEC-MVs can transfer their mitochondrial cargo to neutrophils. Moreover, we demonstrate that these MVs can suppress ROS production from primed neutrophils, consistent with a prior report^50^. Generation of ROS is critical for effective microbial immunity but also plays a significant role in modulating inflammatory responses. The potential consequences of the observed effect on ROS in vivo as well as whether PLY-MVs alter other neutrophil functions are unknown and will require further study. Nonetheless, these findings strongly suggest that AEC can transfer cargo to neutrophils through MVs. Interestingly, a recent study showed that neutrophils can communicate with lung epithelial cells through EVs containing miR-223^51^. This and our current study together introduce a new mechanism of cross-talk between AEC and PMNs via EVs.

Our study has some important limitations. Although we demonstrate that MVs can transfer their mitochondrial cargo to neutrophils, we did not explore the significance of the MV-mitochondrial components on neutrophil functions. Recent studies however, strongly suggest that the mitochondrial content contributes to the biological activity of extracellular vesicles^52^. Puhm et al. recently demonstrated that extracellular vesicles derived from LPS-activated monocytes carry mitochondrial cargo and induce pro-inflammatory signaling in endothelial cells^30^. Vesicles derived from monocytes that were pretreated with inhibitors of mitochondrial stress or mtROS, or from cells with non-respiring mitochondria, had a reduced pro-inflammatory potential^30^. Moreover, mitochondria are important sources of DAMPs, such as mtDNA, cardiolipin, ATP, mROS, N-formyl-peptides^53^. These mitochondrial DAMPs, when released extracellularly target the immune system, including the neutrophils. For example, extracellular mtDNA is a highly inflammatory mediator and a potent stimulus for innate immune activation^54,55^. Importantly, recent studies also suggest that extracellular intact mitochondria can act as danger signals by activating pro-inflammatory signaling in macrophages^56^, interacting with neutrophils to stimulate their adherence to endothelium^31^, and activating neutrophils to produce pro-inflammatory cytokines^57^. Based on these studies, it is reasonable to speculate that the mitochondrial cargo of PLY-induced MVs could play a significant role in their biological effects, a hypothesis that will be the focus of follow-up studies. Another limitation of our current study is that we primarily employed a cancer cell line. Although A549 have been used extensively as a model for alveolar epithelial cells II and to study the effects of pneumolysin^5,10,11,58^, future studies on primary epithelial cells will be needed to confirm these findings.

In the context of acute lung injury, it has been demonstrated that healthy mitochondria from mesenchymal cells can be transferred through EVs to recipient cells (alveolar epithelial cells and macrophages) to promote cytoprotective effects and ameliorate lung injury^59,60^. An intriguing hypothesis is that mitochondria may also be transferred from injured cells to promote pneumonia progression and ALI. Our data support the possibility of this novel injury mechanism. In this study we provide evidence of increased release of MVs from AEC injured with PLY. We also demonstrate that these PLY-induced MVs (a) are rich in mitochondrial cargo that they transfer to neutrophils, and (b) alter neutrophil immune functions. In conclusion, our data indicate that injured alveolar epithelial cells have the potential to regulate innate immune functions through the release of microvesicles.

## Material and methods

### Ethics

Human lung tissues were obtained from adult patients primarily suffering from bronchial carcinoma and undergoing lung resection at thoracic surgery centers. Blood for isolating neutrophils was obtained from healthy volunteers. Written informed consent was received from all patients and volunteers. Ethics approval has been obtained by the local institutional review board (Ethics committee of the Charité-Universitaetsmedizin Berlin, Germany, EA2/079/13 and EA4/032/17, respectively). All animal experiments were approved by institutional (Charité-Universitaetsmedizin Berlin) and governmental authorities [State Office for Health and Social Affairs (Landesamt für Gesundheit und Soziales) Berlin, Germany]. The study was carried out in compliance with the ARRIVE guidelines. All experiments were performed in accordance with relevant guidelines and regulations.

### *S. pneumoniae *strains and pneumolysin

For in vitro studies, D39 wildtype (Spn WT) and PLY-deficient D39Δ*ply* (Spn Δ*ply*) strains were used*.* Bacteria were cultivated in Todd-Hewitt broth (BD, Heidelberg, Germany) supplemented with 0.5% yeast extract (BD, Heidelberg, Germany) to mid-log phase (*A*_600_ = 0.3–0.4) at 37 °C and 5% CO_2_ or grown on Columbia blood agar plates (BD, Heidelberg, Germany). PLY [wild type and non-lytic (NL) PLY] was produced and purified as described^61^.

### Cell culture and treatments

Human alveolar epithelial cells type II (A549 cells, ATCC CCL-185) were cultured in RPMI medium (2 mM glutamine), supplemented with fetal calf serum (FCS; Capricorn Scientific GmbH, Ebsdorfergrund, Germany) at 37 °C and 5% CO_2_. Before experiments, full media (containing FCS) was replaced with plain media, and cells were treated as indicated (PLY, NL-PLY, Spn WT, Spn Δ*ply*) in the Results section. For a set of experiments, cells were pre-treated with inhibitors (BAPTA-AM, Calbiochem) or MitoTempo (Santa Cruz Biotech, USA) prior to PLY treatment. In other experiments, cells were incubated with MitoTracker Deep Red and/or Green (ThermoFisher, Darmstadt, Germany) for 30 min at 37 °C to label mitochondria. Following washing with full media, PBS, and plain media, cells were then incubated with PLY (100 ng/ml, 4 h). After treatments, pictures of cells were captured using the EVOS FLoid cell imaging station (ThermoFisher).

### Ex vivo human lung tissue model

Tumor-free lung tissue was divided into small specimens of the same weight, and incubated for 16–20 h in RPMI 1640 in 12-well dishes^9,62–65^. Prior to treatment, the specimens were transferred to new 12-well dishes and injected with Spn (WT and Δ*ply*) (1 × 10^6^ cfu/ml) or PLY (5 μg/ml) (in plain RPMI, 2 ml volume) as indicated. Conditioned media from each lung specimen was collected and processed.

### Murine model of Spn infection

C57BL/6N female mice (Charles River) were anesthetized with 3 ml/kg of ketamine/xylazine and were intranasally inoculated with 5 × 10^6^ cfu of Spn serotype 3 (PN36) or PBS (controls), as we have described previously^27^. 48 h post-infection, BAL (bronchoalveolar lavage) was obtained by lavaging the lungs with 0.8 ml PBS containing protease inhibitors (Roche Diagnostics GmbH, Germany). BAL fluid (BALF) was obtained after centrifugation at 300 × g for 10 min and stored at − 80 °C until analysis.

### Isolation of microvesicles (MVs)

MVs were isolated as we previously described with some modifications^18^. Briefly, cell and human lung tissue supernatant collected after the indicated treatments was subjected to subsequent centrifugations (500 × g,  5 min and 2,500 × g,  15 min) to eliminate dead cells, apoptotic bodies, and large debris. The final supernatant was centrifuged at 20,500×g for 45 min at 4 °C to pellet the MVs. MVs were then washed in PBS (double filtered), and after careful removal of supernatant, were resuspended in PBS for characterization, quantification, and functional studies. For in vitro experiments, MVs were isolated from dishes containing the same number of cells in the same volume of media. For lung tissue experiments, MVs were isolated from lungs of the same weight in the same volume of media. MVs were also analyzed in BALF after it was further centrifuged at 2,500×g × 10 min.

### Flow cytometry analysis (FACS)

Cells and MVs were analyzed by flow cytometry using a BD FACSCanto II and the BD FACSDiva software to acquire data. For cell analysis, A549 stained with MitoTracker dyes and challenged with PLY as described above, were collected by trypsinization, washed, resuspended in HBSS, and analyzed by FACS. For MV analysis, forward scatter (FSC) and side scatter (SSC) were set at logarithmic gain. No threshold was applied. To set the MV gate (events less than 1 μm), we used 1 μm sizing calibration beads (Sigma-Aldrich). BD CS&T beads consisting of 3-μm bright beads, 3-μm mid, and 2-μm dim polystyrene beads were also run in parallel. In addition, to confirm the gating strategy, fresh human platelets, which are known to have a size of 2–4 μm, were analyzed.

For MV staining, all antibodies/dyes used were from Biolegend unless specified. MVs derived from A549 were stained with annexin V-APC in HBSS+/+(with Ca^2 +^ /Mg^2 +^). As a negative control, MVs were stained with annexin V-APC in PBS containing 5 mM EDTA (annexin V binding to MVs requires calcium, which is removed by the addition of EDTA). To confirm the vesicular nature of the isolated vesicles, MVs were treated with Triton X-100 and analyzed by FACS. A549-MVs were also analyzed after staining with CFSE (Carboxyfluoresceinsuccinimidyl ester) and CellMask Deep Red plasma membrane stain (ThermoFisher). For this, MVs were incubated at 37 °C with CFSE or CellMask dyes for 20 min, and washed in PBS before analysis. CSFE and CellMask solutions were centrifuged at high-speed for 5 min before added to MVs. As a negative control, samples (PBS) that contain the dyes but not MVs were processed in parallel. For MVs from MitoTracker-labeled cells, MVs were isolated, resuspended in PBS and analyzed by FACS. MVs from human lung tissue or in BALF were analyzed after double staining with annexin V and anti-human or anti-mouse EPCAM-Alexa 488, respectively, in HBSS+/+. As a negative control, MVs were stained with annexin V-APC in PBS containing 5 mM EDTA and the corresponding isotype control antibody. All buffers used for MV analysis were double filtered (0.22 μm). For MV quantification, 5 μl of CountBright absolute counting beads (ThermoFisher) were added in each tube before analysis. To assess background signals, several negative controls were used (a) buffers with antibodies alone were analyzed to evaluate the background fluorescence of antibodies, (b) plain media after staining with antibodies to detect artifacts in the media, (c) single-stained MVs, (d) buffers containing only CFSE, CellMask, or MitoTracker dyes. FACS data were analyzed using the FCS Express 6 Flow cytometry software (Denovo Software, Glendale, CA).

### Protein measurement

Quantification of isolated MVs was also done using a NanoDrop 2000 spectrophotometer (ThermoFisher). MV suspensions were measured at 280 nm for protein quantification.

### Immunoblotting

MV lysates (MVs isolated from the same number of cells) prepared in Laemmli buffer were subjected to SDS-electrophoresis using 4–20% precast gels (Genscript, Piscataway, NJ). After protein transfer, membranes were incubated overnight with indicated antibodies, previously characterized (Santa Cruz Biotechnology)^66–68^. After incubation with secondary antibodies-HRP conjugated (Santa Cruz Biotechnology), proteins were detected with the Pierce ECL (ThermoFisher) or Amersham ECL Prime (Sigma) western blotting reagents. Films were scanned and pictures were processed using Image J (NIH). Brightness and contrast were adjusted at the same levels for samples in the sample blot. There was no grouping of blots cropped from different parts of the same blot. Unedited blots are presented in Supplementary Figure S2.

### Confocal microscopy

CFSE-labeled MVs were added in 8 well μ-slides, ibiTreat (Ibidi, Martinsried, Germany). Following a short centrifuge of the slide, MVs were immediately visualized using an LSM 780 confocal laser-scanning microscope driven by Zen 2012 software. 1 μm latex beads were also visualized for comparison. Images were processed using the software Zen2.3SP1 (Carl Zeiss).

### Neutrophil isolation

Human neutrophils were isolated from whole blood by a 3% dextran sedimentation step (Dextran T500, Carl Roth GmbH, Germany) followed by centrifugation over a 1.077 g/ml Pancoll solution (PAN-Biotech). Cell viability was evaluated by trypan blue exclusion, and purity (> 95%) was verified by flow cytometry. After isolation, neutrophils were resuspended in HBSS +/+, quantified, and used for experiments as indicated.

### MitoTracker-positive microvesicle uptake by neutrophils

Neutrophils were incubated with MVs isolated from PLY-or PBS (Control; Ctr)- treated MitoTracker Deep Red (APC)-labeled A549 (4 × 10^6^ cells) at 37 °C or 4 °C. After 1 h of incubation, neutrophils were washed, stained with CD11b (Brilliant Violet 510, Biolegend) and 7-AAD (Biolegend), and analyzed by FACS. For each sample, an average of 5000 cells was analyzed and the percentage of CD11b + /7-AAD−/APC + cells was recorded.

### Reactive Oxygen Species (ROS) production

Neutrophils were incubated in a 96-well plate before luminol reagents were added to each well (50 μM luminol and 1.2 U/ml HRP substrate). After a short incubation, neutrophils were primed with low doses of PMA (0.5 nM) and 5 min later, PLY-MVs (5 μg of protein isolated from 4 × 10^6^ cells) were added. MVs from control cells were also tested in parallel. Luminescence was recorded [SpectraMax L (Molecular Devices, USA)] over a period of 1 h (1 min interval). Area under the curve was calculated for each condition.

### Statistical analyses

Results are expressed as mean ± s.e.m. Statistical analysis was performed using the Graphpad Prism 8 software. Data were tested for normal distribution by Shapiro–Wilk test. T-test (paired or un-paired) and one-way ANOVA were used to compare means between groups. Mann–Whitney test was used to compare non-parametric data sets (specified in the respective figure legend). A p-value < 0.05 was considered statistically significant.

## Supplementary Information

Supplementary Information.

## Data Availability

The datasets generated during and/or analyzed during the current study are available from the corresponding author on reasonable request.
