# Supplementary material for: Microvesicles released from pneumolysin-stimulated lung epithelial cells carry mitochondrial cargo and suppress neutrophil oxidative burst
Source: Sci Rep. 2021 May 5;11:9529. doi: 10.1038/s41598-021-88897-y (PMC8100145; doi:10.1038/s41598-021-88897-y)
Supplement: Supplementary file 1 — Supplementary Information. [file 41598_2021_88897_MOESM1_ESM.pdf]

Microvesicles released from pneumolysin-stimulated lung epithelial cells carry mitochondrial cargo and suppress neutrophil oxidative burst

Letsiou E.[1,2*], Teixeira Alves LG.[1], Fatykhova D.[1], Felten M.[1], Mitchell TJ.[3], Mueller-Redetzky H.[1], Hocke AC.[1,4], Witzenrath M.[1,4]

[1]Charité – Universitätsmedizin Berlin, corporate member of Freie Universität Berlin and Humboldt-Universität zu Berlin, Division of Pulmonary Inflammation, and Department of Infectious Diseases and Respiratory Medicine, Charitéplatz 1, 10117 Berlin, Germany.

[2]Division of Pulmonary, Critical Care, Sleep, and Allergy, University of Illinois at Chicago, 60612 Chicago, Illinois, USA.

[3]Institute of Microbiology and Infection, College of Medical and Dental Sciences, University of Birmingham, B152IT, Edgbaston UK.

[4]German Center for Lung Research (DZL)

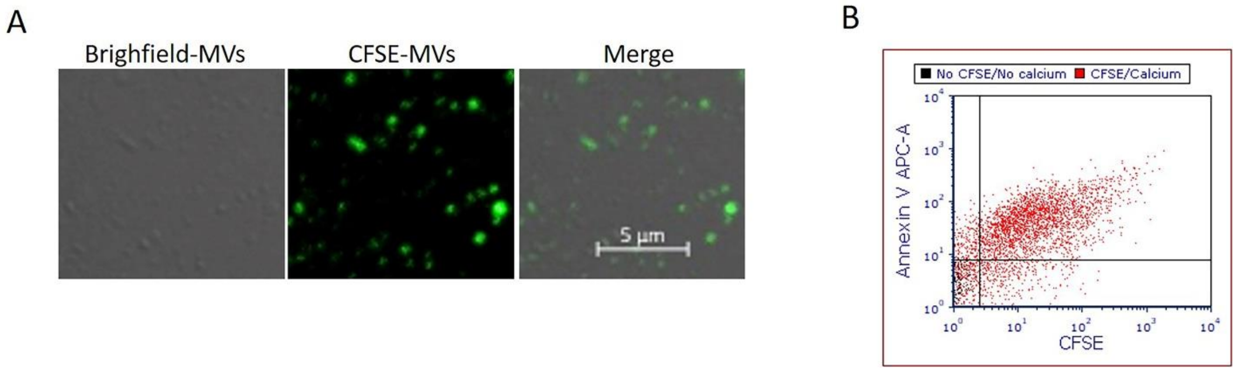

Supplementary Figure 1. CFSE-labeled microvesicles. MVs isolated from PLY (100 ng/ml, 4 hours)-treated A549 were labeled with CFSE. **a.** CFSE-MVs were analyzed by confocal microscopy. Brightfield (left panel), fluorescence (middle panel), and merged (right panel) of the CFSE-labeled MVs were taken. **b.** Representative FACS dot plot of CFSE-MVs stained with annexin V (APC) in the presence (Red dots) or absence of calcium (black dots in the lower left quadrant; negative staining).

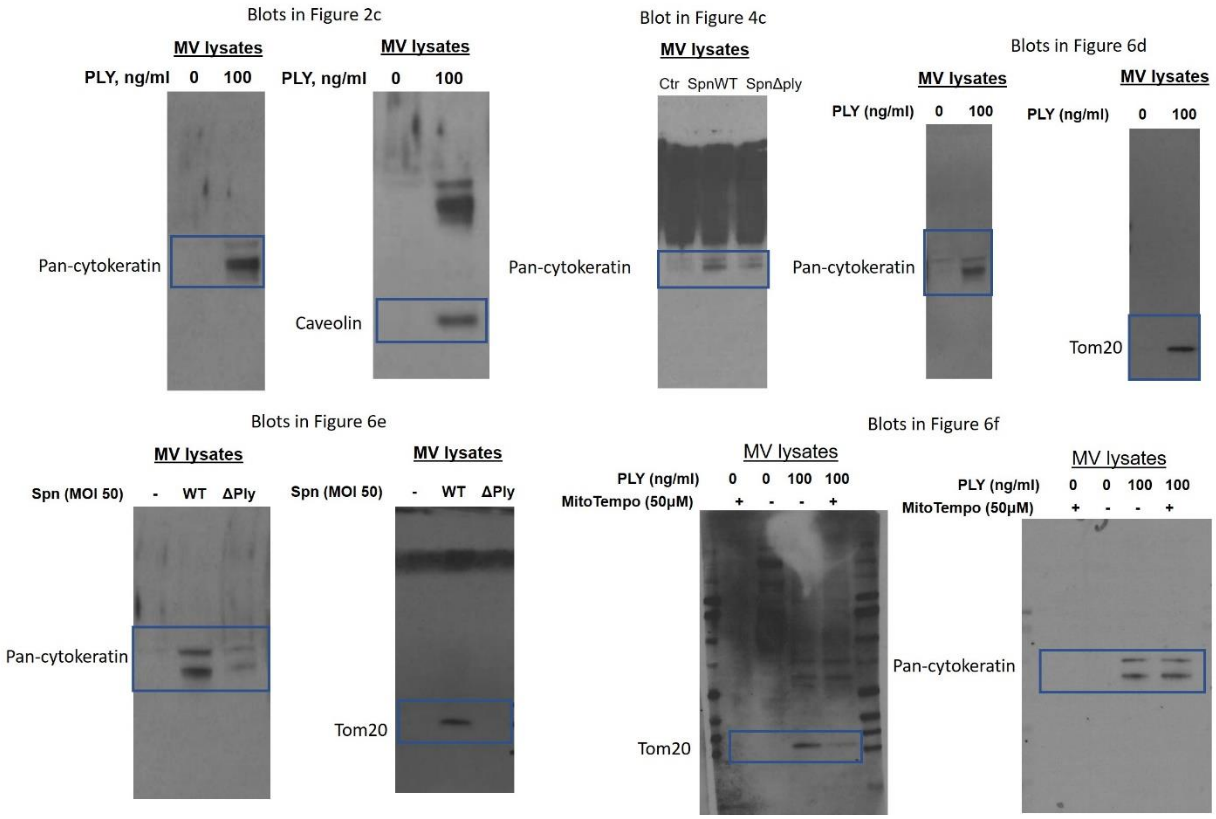

Supplementary Figure 2. Full length blots.
